# Twice or once a day? Filgrastim dosing schedule for peripheral hematopoietic stem cells mobilization

**DOI:** 10.1016/j.htct.2025.103738

**Published:** 2025-02-15

**Authors:** Mehmet Bakırtaş, Bahar Uncu Ulu, Tuğçe Nur Yiğenoğlu, Semih Başcı, Ali Kılınç, Tahir Darçın, Fatma Nurbüke Şarkışla, Derya Şahin, Nuran Ahu Baysal, Dicle İskender, Merih Kızıl Çakar, Mehmet Sinan Dal, Fevzi Altuntaş

**Affiliations:** a Department of Hematology, Republic of Turkey Ministry of Health Tekirdağ Dr. İsmail Fehmi Cumalıoğlu City Hospital, Tekirdağ, Turkey; b Department of Hematology and Bone Marrow Transplantation, Dr. Abdurrahman Yurtaslan Ankara Oncology Training and Research Hospital, University of Health Sciences, Ankara, Turkey; c Department of Apheresis, Dr. Abdurrahman Yurtaslan Ankara Oncology Training and Research Hospital, University of Health Sciences, Ankara, Turkey; d Department of Internal Medicine, Dr. Abdurrahman Yurtaslan Ankara Oncology Training and Research Hospital, University of Health Sciences, Ankara, Turkey

**Keywords:** Filgrastim, Hematopoietic stem cell mobilization, G-CSF, Posology

## Abstract

**Introduction:**

Granulocyte colony-stimulating factor (G-CSF) is the most prevalently used growth factor for peripheral blood hematopoietic stem cell (HSC) mobilization. Most centers split the granulocyte colony-stimulating factor in two daily doses, whereas some centers administer one dose per day. This study aims to investigate the effect of the filgrastim dosing schedule on the quantity of hematopoietic stem cells collected after mobilization in healthy donors.

**Methods:**

A total of 251 healthy donors mobilized in our center were included in the study. Mobilization was either once a day (filgrastim 1 × 10 mg/kg/day) or twice a day (filgrastim 2 × 5 mg/kg/day).

**Results:**

White blood cell and peripheral CD34^+^ cell numbers were significantly higher in the Twice-a-day Group on the fifth day compared to the Once-a-day Group. No statistically significant difference was shown between the two groups regarding the number of CD34^+^ cells collected on the first apheresis day or the number of apheresis procedures needed to achieve the targeted number of CD34^+^ cells.

**Conclusion:**

This study revealed that one daily dose of 10 mg/kg filgrastim is as effective as administering the same dose split on two days for an adequate amount of CD34^+^ cells in healthy donors.

## Introduction

Allogeneic stem cell transplantation (Allo-SCT) is a potentially curative therapy for a range of benign and malignant hematologic disorders. Since the mid-1990s, granulocyte colony-stimulating factor (G-CSF) has been used to mobilize peripheral blood (PB)-derived hematopoietic stem cells (HSCs) in healthy donors for Allo-SCT.^1^^,^^2^ Nowadays, PB-derived HSCs are used in about 75 % of Allo-SCTs.^3^ When it comes to malignant hematologic disorders, PB-derived HSCs are the preferred choice due to their shorter engraftment durations, easier and more tolerable collection process, and higher graft-versus-leukemia/lymphoma effects compared to HSCs derived from bone marrow (BM).^4^ The effectiveness of an Allo-SCT relies on the infusion of a sufficient number of HSCs.^5^^,^^6^ HSCs normally constitute 1–3 % of nucleated BM cells, and HSCs are physiologically released from the BM into the PB. With this process, HSCs make up a mere 0.01–0.05 % of all nucleated cells in the PB.^7^, ^8^, ^9^

G-CSF is the most commonly employed cytokine for PB-HSC mobilization. The stimulation of HSC mobilization with G-CSF can increase HSCs to 0.5–1 % in the PB.^9^ HSCs exhibit a range of surface receptors, including chemokine receptor type 4 (CXCR4) and type 2 (CXCR2), as well as the CD44 and CD62 L surface glycoproteins.^10^ The BM stroma contains stromal cell-derived factor 1 (SDF-1), vascular cell adhesion molecule (VCAM-1), KIT ligand, P-selectin glycoprotein ligand, and hyaluronic acid, all of which are ligands for HSC adhesion molecules.^10^^,^^11^ G-CSF suppresses the interaction between SDF-1 and CXCR4, leading to a reduction in the attachment of HSCs to various receptors in the BM stroma.^12^^,^^13^ As progenitor and precursor cells, HSCs express the cell surface marker antigen CD34, which is an indicator of PB-HSC mobilization efficiency.^10^

The correlation between the dosage of G-CSF and the number of CD34^+^ cells collected has been demonstrated in both healthy donors and cancer patients.^14^, ^15^, ^16^, ^17^ The European Blood and Bone Marrow Transplantation Group (EBMT) recommends administering 10 mg/kg/day G-CSF for PB-HSC mobilization in healthy donors.^18^

Several centers administer G-CSF in two doses (2 × 5 mg/kg/day), while other centers administer G-CSF once a day (1 × 10 mg/kg/day).^19^^,^^20^ The administration of G-CSF twice a day (2 × 5 mg/kg/day) has been reported to be superior to once-a-day (1 × 10 mg/kg/day) administration, although there are studies indicating that there is no difference between the two methods regarding the amount of CD34^+^ cells obtained following PB-HSC mobilization.^21^^,^^22^

This single-center, retrospective study aims to investigate the effect of the filgrastim dosing schedule on the quantity of HSCs collected after mobilization in healthy donors.

## Materials and methods

### The donors

This study included healthy donors aged ≥18 years who were mobilized with filgrastim. PB HSC mobilization was performed at our center. Data regarding the donors’ age, gender, weight, body mass index, filgrastim dose schedule, white blood cell (WBC) count, the number of peripheral CD34^+^ stem cells, the number of CD34^+^ cells harvested, the volume of the product, the processed blood volume, and the duration and number of apheresis procedures were recorded. All the collected data were analyzed retrospectively. Medical history and physical examinations were performed both on the first evaluation day before starting G-CSF and on the first day of apheresis. Donors with any organ dysfunction or infectious disease were excluded.

### Hematopoietic stem cell mobilization

All donors were mobilized using G-CSF only. They received a total dose of 10 mg/kg/day of filgrastim for 4 of 5 days subcutaneously. The healthy donors were divided according to the filgrastim dosing schedule. They were separated into Once-a-day (1 × 10 mg/kg/day) and Twice-a-day (2 ×5 mg/kg/day, given at 7:00 am and 7:00 pm) Groups. On the fourth day, filgrastim was omitted in donors whose WBC counts were ≥75 × 10^9^/L and the filgrastim dose was reduced to half for donors whose WBC count was ≥50 × 10^9^/L. On the fifth day, HSCs were harvested using the same apheresis technique after equal time intervals (2 h) following the administration of the filgrastim dose in the morning in both the Once-a-day and Twice-a-day Groups. If the quantity of CD34^+^cells collected did not meet the desired goal, the administration of filgrastim was continued, and the apheresis procedure was repeated the following day. The apheresis procedure was performed using a continuous flow cell separator (Fresenius Kabi Com.tec, Germany). For each apheresis session, two to two and a half times the donors' total blood volume was processed at a flow rate of 50 to 60 mL/min.

### Ethical considerations

This study was conducted in accordance with the criteria established by the Declaration of Helsinki. Every donor provided informed written consent prior to the collection of stem cells. This study received ethics approval from the Ethics Committee of the University of Health Sciences, Dr. Abdurrahman Yurtaslan Ankara Oncology Training and Research Center.

### Statistical analyses

Statistical analysis was conducted using IBM SPSS Statistics software (version 21). The data are summarized using descriptive statistics. Categorical data are represented as ratios, while numerical data are represented using the median and mean ± standard deviation. The Mann-Whitney U test was used to evaluate continuous variables between groups, while the chi-Square test was used to analyze categorical variables. *p*-values <0.05 were considered statistically significant.

## Results

Two hundred and fifty-one healthy donors were allocated to the Once-a-day Group (filgrastim 1 × 10 mg/kg/day; *n* = 121) or the Twice-a-day Group (filgrastim 2 × 5 mg/kg/day; *n* = 130) according to filgrastim dosing schedules. The median age was 30 years (range: 18–65 years) in the entire cohort. The median age was 30 years (range: 19–52 years) in the Once-a-day Group and 33 years (range: 18–65 years) in the Twice-a-Day group. Both groups were similar regarding gender (*p*-value = 0.227) and body weight (*p*-value = 0.093) characteristics. The WBC count was 37.5 × 10^9^ cells/L (range: 21.9–76 × 10^9^ cells/L) and 43 × 10^9^ cells/L (range: 26.36–62.33 ×10^9^ cells/L) in the once-a-day and twice-a-day Groups, respectively. The median PB CD34^+^ cell level on the 5th day of G-CSF was 69.1 cells/µL (range: 8.1–292 cells/µL) and 79.5 cells/µL (range: 8.5–232.6 cells/µL) in the Once-a-day and Twice-a-day Groups, respectively. WBC count and PB CD34^+^cell levels on the fifth day were significantly higher in the Twice-a-day Group compared to the Once-a-day Group (*p*-value = 0.043 and *p*-value = 0.002, respectively). The median number of CD34^+^cells collected was 7.4 × 10⁶ cells/kg (range: 2.08–33 × 10⁶ cells/kg) and 7.9 × 10⁶ cells/kg (range: 2.06–49 ×10⁶ cells/kg) on the first apheresis day in the Once-a-day and Twice-a-day Groups, respectively.

There was no statistically significant difference observed between the two groups in terms of the quantity of CD34^+^stem cells collected on the initial day of apheresis, as well as the median number of CD34^+^cells in the product on the first day of apheresis (Table 1).

**Table 1:** Variables of donors who received granulocyte colony-stimulating factor (filgrastim) twice per day (2 × 5 mg/kg/day) or once per day (1 × 10 mg/kg/day).

Parameter	Filgrastim	Filgrastim	*p*-value
	2 × 5 mg/kg/day	1 × 10 mg/kg/day	
Gender (female/male) - n	27/103	33/88	0.227
Donor's Weight (kg) - median	80 (54–120)	76 (49–135)	0.093
BMI (kg/m^2^) - median	26.1 (17.6–34.9)	24.8 (17.8–39.4)	0.182
WBC (x 10^6^ cells/µL) - median	43.00 (26.36–62.33)	37.50 (21.90–76.00)	**0.043**^a^
Peripheral CD34^+^ cell number on the 5th G-CSF day (µL) - median	79.5 (8.5–232.6)	69.1 (8.1–292)	**0.002**^a^
CD34^+^ cell collected on the first apheresis day (10^6^/patient's kg) - median	7.9 (2.06 – 49.00)	7.4 (2.08–33.00)	0.441
Product CD34^+^ cells collected on the first apheresis day (µL) - median	1145 (371- 4068)	1391 (246–10,007)	0.322
Total blood volume (mL) - median	5250 (3120–7840)	5460 (3250–8750)	0.1
Processed blood volume (mL) - median	11,485 (6890–18,250)	12,320 (6500–18,700)	0.07
Apheresis duration (min) - median	240 (150–400)	240 (150–330)	0.44
Product volume (mL) - median	332.5 (150–540)	340 (190–540)	0.43

BMI: body mass index; WBC: White blood cell count; G-CSF: granulocyte colony-stimulating factor.

a *p*-value < 0.05.

While 18 donors (15 %) in the Once-a-day Group required a second apheresis procedure to reach the targeted quantity of CD34^+^cells (5 × 10⁶/kg), 26 donors (20 %) in the Twice-a-day Group required a second apheresis procedure. There was no significant difference between the two groups regarding the number of apheresis procedures needed to collect the targeted quantity of total CD34^+^stem cells (*p*-value = 0.079). No significant differences were observed between the two groups in terms of total blood volume, processed blood volume, apheresis duration, or product volume (Table 1).

## Discussion

G-CSF is widely used for peripheral HSC mobilization; however, the ideal method for HSC mobilization is still being researched. Most studies were conducted in the late 1990s and early 2000s. To our knowledge, this is the only study conducted in the last decade that addresses the optimal filgrastim dosing schedule and has the largest donor cohort.

The major difference between the studies conducted lies in the timing of the leukapheresis procedure. After the initial G-CSF administration, leukapheresis was started on the fifth day of mobilization. Although the majority of facilities administered G-CSF once daily, some suggested splitting the doses into two and administering them at 12-hour intervals.^19^, ^20^, ^21^, ^22^ According to this study, WBC counts and PB CD34^+^ cell levels on the fifth day were significantly higher in the twice-a-day Group compared to the Once-a-day Group. However, there was no statistically significant difference between the two groups in terms of the quantity of CD34^+^HSCs collected on the initial day of apheresis or the number of apheresis procedures performed. PB CD34^+^ levels did not impact mobilization success, and once-a-day administration may be more convenient for healthy outpatient donors.

Regarding the use of plerixafor prior to apheresis, none of the donors in this study received plerixafor. This study center primarily mobilizes healthy donors with G-CSF alone, with plerixafor typically being reserved for autologous settings or cases where initial mobilization efforts fail. This protocol may limit direct comparisons with studies utilizing plerixafor but provides insights into G-CSF-only mobilization outcomes.

Optimized PB HSC mobilization is important for allogeneic donors, as it results in fewer apheresis procedures and less exposure to G-CSF. For patients, a higher number of infused hematopoietic progenitor cells leads to quicker engraftment, reduces complications, and improves survival rates.^23^ To avoid mobilization failure, it is essential to identify the factors that negatively affect the quantity of CD34^+^cells collected. Characteristics such as older age, female gender, and iron deficiency have been recognized as detrimental factors for PB HSC mobilization.^24^, ^25^, ^26^

In this study, the Twice-a-day Group had a slightly higher median age compared to the Once-a-day Group. While this difference was not statistically significant, it is important to consider age-related impacts on mobilization success. Previous studies have indicated that older age may be associated with reduced mobilization efficiency, although this effect was not strongly observed in the current cohort.

The European Blood and Marrow Transplantation Group (EBMT) recommends the administration of 10 mg/kg/day G-CSF for PB HSC mobilization in healthy donors, but the optimal schedule has yet to be established.^18^ The pharmacokinetics of subcutaneous G-CSF in healthy donors and cancer patients show a maximum serum concentration within 2 ± 8 h, and due to the short elimination half-life of G-CSF (approximately 3 ± 4 h), thus a twice-daily schedule may improve CD34^+^cell yield.^27^^,^^28^ Although previous studies have shown that administering G-CSF twice daily (2 × 5 mg/kg/day) was superior to once-daily administration (1 × 10 mg/kg/day) for mobilizing CD34^+^cells, other studies have found no significant difference between the two methods in terms of PB HSC mobilization efficiency.^21^^,^^22^ In a study by Komeno et al., once-daily filgrastim administration was found to be as effective as twice-daily administration for CD34^+^cell mobilization in healthy donors.^29^ On the contrary, a prospective randomized controlled study conducted by Kroger et al. demonstrated that more CD34^+^cells were obtained with twice-daily administration (2 × 5 mg/kg/day) compared to once-daily administration (1 × 10 mg/kg/day).^22^ Another study by Anderlini et al. showed no difference between the two methods in terms of PB HSC mobilization efficiency in healthy donors.^21^ In the current study, WBC and PB CD34^+^ cell levels on the fifth day were significantly higher in the Twice-a-day Group compared to the Once-a-day Group. However, no statistically significant difference was observed between the two groups in terms of the number of CD34^+^HSCs collected on the first day of apheresis or the number of apheresis procedures required to achieve the targeted number of CD34^+^cells.

It is worth noting that the Twice-a-day Group had a lower processed blood volume compared to the Once-a-day Group. Although this difference was not statistically significant, it may suggest that the higher CD34^+^cell levels observed in the Twice-a-day Group allowed for a similar stem cell yield with a lower processed volume. This observation highlights a potential advantage of the twice-daily schedule, which could be beneficial in terms of donor comfort and procedure efficiency.

While the twice-daily filgrastim schedule is often considered in terms of drug pharmacology, the present study shows that once-daily filgrastim administration does not affect the ability to reach the targeted number of PB CD34^+^cells. Considering healthy donors, one injection per day seems to be both convenient and sufficient for ease of administration. The retrospective design of this study and the lack of data on side effects associated with different filgrastim dosing schedules in healthy donors are among its limitations.

While this study provides valuable insights into the potential equivalence of once-daily and twice-daily filgrastim dosing for PB HSC mobilization in healthy donors, several limitations warrant consideration:

• Retrospective studies are inherently susceptible to bias due to the inability to control for confounding variables. Prospective randomized controlled trials would provide stronger evidence.
• This study lacks information on long-term outcomes of transplant recipients who received HSCs mobilized with different dosing schedules. Evaluating factors such as engraftment, graft-versus-host disease incidence, and overall survival could provide a more comprehensive understanding.
• Results may not be generalizable to other centers with different patient populations, mobilization protocols, or apheresis procedures. Multicenter studies are needed for broader applicability.
• The study does not explore potential differences in side effects between the two dosing schedules, which could be relevant for donor comfort and compliance.
• The study focused on healthy donors, excluding individuals with underlying medical conditions or specific donor characteristics that might influence mobilization efficiency. Further research is needed to assess the generalizability of findings to broader donor populations.

## Conclusion

• This study compared the efficacy of administering 10 mg/kg filgrastim once or twice daily for PB-HSC mobilization in healthy donors.
• Although the WBC count and PB CD34^+^ cell levels on the fifth day were significantly higher in the Twice-a-day Group, there was no statistically significant difference in the number of CD34^+^ HSCs collected during the first apheresis or in the number of apheresis procedures needed to reach the target CD34^+^ cell count.
• This suggests that once-daily filgrastim administration may be as effective as twice-daily administration for healthy donors in mobilizing sufficient CD34^+^ cells.
• The study findings support the potential convenience and practicality of once-daily filgrastim administration for outpatient donors.
• However, limitations such as the retrospective design and the lack of side effect data necessitate further research for confirmation and broader applicability.

In conclusion, this study highlights the potential equivalence of once-daily and twice-daily filgrastim dosing for PB-HSC mobilization in healthy donors. However, considering the limitations, further research with more robust designs and a broader scope is crucial to solidify these findings and optimize mobilization strategies for various donor populations.

## Author contributions

Design of the study: M.B., B.U.U., T.N.Y., F.A., M.S.D.

Data gathering: S.B., A.K., N.A.B., F.N.Ş.

Searching the literature: M.B., T.D., B.U.U, M.K.Ç., D.İ., D.Ş.

## Conflicts of interest

The authors declared no potential conflicts of interest concerning the research, authorship, and/or publication of this article.
